# Clinical Audit of Initial Plasma Monitoring of Clozapine and Lithium Across Inpatient Psychiatric Wards

**DOI:** 10.1192/bjo.2026.11812

**Published:** 2026-06-30

**Authors:** Ei Shwe Sin Oo, Samuel Adenle

**Affiliations:** 1 Tees, Esk and Wear Valley NHS Foundation Trust, Durham, United Kingdom; 2 Tees, Esk and Wear Valley NHS Foundation Trust, Middlesbrough, United Kingdom

## Abstract

**Aims:**

To verify whether clozapine and lithium plasma levels were monitored in accordance with trust guidelines in inpatient psychiatric settings.

**Methods:**

A retrospective audit was conducted across eight inpatient wards.

Fifteen inpatients prescribed clozapine and/or lithium were included.

The audit period covered December 2024 to March 2025.

Data sources included clinical notes, WebICE, EPMA, and clozapine monitoring report form

**Standards and Criteria**

**Clozapine:** Plasma levels taken immediately before the morning dose (for twice-daily dosing) or 10–12 hours post-dose (for once-daily night dosing), after at least three days on a stable dose.

**Lithium:**Plasma levels taken 5–7 days after initiation, sampled 12–14 hours post-dose, with weekly monitoring until stable therapeutic levels are achieved.

**Target compliance:** 100%.

**Results:**

**Clozapine (n=6):** All patients had plasma levels checked after at least three days on a stable dose. Four patients (66.67%) had samples taken within the recommended post-dose time window. For two patients (33.33%), compliance could not be confirmed due to missing uploaded laboratory reports. Two patients were within the therapeutic range, while four were outside the target range. Dose adjustments were made for two patients; no changes were required for two due to good clinical response or increased cigarette use. Repeat plasma levels were obtained where clinically indicated.

**Lithium (n=9):** Seven patients (77.8%) had lithium levels checked within 5–7 days of initiation. Two patients (22.2%) did not meet the recommended initiation timing. Two patients had samples taken outside the recommended 12–14-hour post-dose window. Eight patients had plasma levels outside the therapeutic range, prompting dose adjustments and repeat testing. One patient had a therapeutic lithium level and required no dose adjustment.

**Conclusion:**

**Summary:** Only 66.67% of patients on clozapine or lithium were fully monitored in line with trust guidelines. Missing clozapine monitoring forms limited the ability to assess compliance. Inconsistent plasma sampling times were identified.

**Recommendations and Action:** Plan Improve staff awareness by displaying trust guidelines on wards and providing targeted teaching sessions. Educate patients on the importance of adherence to plasma monitoring schedules. Emphasise the importance of uploading clozapine monitoring forms and assign clear accountability. Work with IT services to address technical issues related to report uploads.

**Conclusion:**

This audit demonstrated partial compliance with trust guidelines for clozapine and lithium plasma monitoring. Improved documentation practices and targeted staff education are recommended to enhance patient safety. A re-audit is planned to assess the impact of these interventions

